# Acetylenic Synthetic Betulin Derivatives Inhibit Akt and Erk Kinases Activity, Trigger Apoptosis and Suppress Proliferation of Neuroblastoma and Rhabdomyosarcoma Cell Lines

**DOI:** 10.3390/ijms222212299

**Published:** 2021-11-14

**Authors:** Sylwia K. Król, Ewa Bębenek, Magdalena Dmoszyńska-Graniczka, Adrianna Sławińska-Brych, Stanisław Boryczka, Andrzej Stepulak

**Affiliations:** 1 Department of Biochemistry and Molecular Biology, Faculty of Medicine, Medical University of Lublin, Chodźki 1, 20-093 Lublin, Poland; magdalena.dmoszynska-graniczka@umlub.pl (M.D.-G.); andrzej.stepulak@umlub.pl (A.S.); 2 Department of Organic Chemistry, Faculty of Pharmaceutical Sciences in Sosnowiec, Medical University of Silesia, Jagiellońska 4, 41-200 Sosnowiec, Poland; ebebenek@sum.edu.pl (E.B.); boryczka@sum.edu.pl (S.B.); 3 Department of Cell Biology, Faculty of Biology and Biotechnology, Institute of Biological Sciences, Maria Curie-Sklodowska University, Akademicka 19, 20-033 Lublin, Poland; adrianna.slawinska-brych@poczta.umcs.lublin.pl

**Keywords:** acetylenic synthetic betulin derivatives, ADMET, betulin, chemotherapy, cisplatin, druglikeness, neuroblastoma, pediatric cancers, rhabdomyosarcoma, temozolomide

## Abstract

Neuroblastoma (NB) and rhabdomyosarcoma (RMS), the most common pediatric extracranial solid tumors, still represent an important clinical challenge since no effective treatment is available for metastatic and recurrent disease. Hence, there is an urgent need for the development of new chemotherapeutics to improve the outcome of patients. Betulin (Bet), a triterpenoid from the bark of birches, demonstrated interesting anti-cancer potential. The modification of natural phytochemicals with evidenced anti-tumor activity, including Bet, is one of the methods of receiving new compounds for potential implementation in oncological treatment. Here, we showed that two acetylenic synthetic Bet derivatives (ASBDs), EB5 and EB25/1, reduced the viability and proliferation of SK-N-AS and TE671 cells, as measured by MTT and BrdU tests, respectively. Moreover, ASBDs were also more cytotoxic than temozolomide (TMZ) and cisplatin (cis-diaminedichloroplatinum [II], CDDP) in vitro, and the combination of EB5 with CDDP enhanced anti-cancer effects. We also showed the slowdown of cell cycle progression at S/G_2_ phases mediated by EB5 using FACS flow cytometry. The decreased viability and proliferation of pediatric cancers cells after treatment with ASBDs was linked to the reduced activity of kinases Akt, Erk1/2 and p38 and the induction of apoptosis, as investigated using Western blotting and FACS. In addition, in silico analyses of the ADMET profile found EB5 to be a promising anti-cancer drug candidate that would benefit from further investigation.

## 1. Introduction

Cancer still remains a leading public health issue and is a major cause of premature death worldwide, resulting in multiple severe social and economic problems. Moreover, the global tumor burden is predicted to increase within next decades [1,2].

Neuroblastoma (NB) is one of the most frequent solid cancers in infants and young children, representing approximately 8–10% of all childhood malignancies [3,4]. NB, as an embryonal tumor originated from stem cells of the neural crest, may arise within the sympathetic nervous system in the neck, chest, abdomen or pelvis. Therefore, NB remains a complex disease with diverse clinical and histopathological manifestations [4,5]. Recent advanced high-throughput study and large-scale profiling showed several genome, epigenome and transcriptome abnormalities determining high biological and clinical heterogeneity and variability of pediatric neuroblastomas [5]. Prognoses for patients with NB are quite variable—from spontaneous regression mainly in infants ≤18 months, to aggressive disease, characterized by distant metastases and resistance to standard treatment modalities in older children [3]. Current treatment options for children with NB are local-control surgical resection, radiotherapy, an immunotherapeutic approach using monoclonal antibodies [5] and multidrug chemotherapy with irinotecan, topotecan, vincristine-irinotecan [6,7,8] and cisplatin (cis-diaminedichloroplatinum[II], CDDP) [9,10,11]. However, no significant progress has been achieved in terms of survival rates for patients with advanced or metastatic NB.

Pediatric soft-tissue sarcomas represent a very heterogeneous group of malignant tumors of mesenchymal stem or progenitor cell origin, accounting for 7% of all childhood cancers (between 2 to 6 and 10 to 18 years old) [12]. Among them, the most common type (nearly 40%) is rhabdomyosarcoma (RMS), generally localized in the head, neck and genitourinary system (embryonal RMS, mostly in younger children) or trunk regions (alveolar RMS, in older children) [13]. The significant histological and biological complexity of RMS was evidenced by whole-genome and transcriptome sequencing of rhabdomyosarcoma cells that revealed frequent genetic aberrations such as chromosomal translocations, allelic loss, gene mutations and fusions. Although the majority of children with localized disease may benefit from multi-modal therapy that improves 5-year survival rates by up to approximately 70%, the outcomes of patients with high-risk metastatic or recurrent RMS are still unsatisfactory [14]. Currently available standards of treatment include surgery, combination chemotherapy (VAC, vincristine–actinomycin D-cyclophosphamide and IVA, ifosfamide-vincristine-actinomycin D in the USA and Europe, respectively) and/or radiation. However, therapeutic protocol has not changed prominently since the 1980s [13].

To conclude, there is still a strong need for novel alternative types of chemotherapeutic agents which will overcome cancer cells’ resistance, augment treatment effectiveness and reduce heavy adverse reactions to improve the outcome of pediatric cancer patients.

Betulin (Bet, 3-lup-20(29)-ene-3*β*, 28-diol) is a pentacyclic lupane-type triterpenoid which naturally occurs abundantly in the outer bark of birch trees (*Betula*, *Betulaceae* family) [15]. Bet has been found to demonstrate a broad spectrum of biological and pharmacological activities, among which, its chemopreventive and anti-tumor activities attract the most attention [16,17]. Several in vitro studies have evidenced the significant inhibition of cancer cells’ viability/survival, growth and proliferation [18,19,20,21,22,23,24,25,26,27,28,29,30,31,32], migration [18], angiogenesis [24,31,33,34], as well as perturbation in cell cycle progression [31,35] and the induction of apoptosis [18,20,23,24,29,31,36,37] after treatment with Bet. What is more, its promising activity has also been evidenced in animal models [24,31,34,38,39]. As a result of its multiple anti-cancer activities, selective cytotoxicity towards cancer cells and relatively low toxicity against normal cells [18,25,40,41,42,43], Bet has also been utilized as a precursor compound for the synthesis of numerous novel derivatives. The structure of the Bet molecule enables several chemical modifications and consequently, many of its derivatives with improved biological and pharmacological properties can be synthesized for potential implementation into clinical oncology [17,44,45].

A series of Bet derivatives bearing an alkyne moiety at carbon C-3 and/or C-28 (acetylenic synthetic Bet derivatives, ASBDs) were synthesized, and the procedure as well as structural analysis and comprehensive chemical characterization were published [25]. In this study, we aimed to investigate the anti-tumor potential of two ASBDs, 28-*O*-propynoylbetulin (EB5) and 28-*O*-propargyloxycarbonylbetulin (EB25/1), in both NB and RMS pediatric cancers in vitro. Additionally, we also calculated physicochemical parameters of the analyzed ASBDs and predicted their druglikeness, medicinal chemistry friendliness and ADMET profile (absorption, distribution, metabolism, excretion and toxicity) in silico. Our findings support the rationale for further studies on EB5 derivatives and validation in animal models for prospective development as a new chemotherapeutic for clinical practice.

## 2. Results

**Figure 1:**
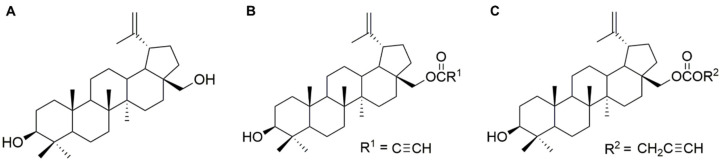
Chemical structure of betulin (Bet) and its acetylenic synthetic derivatives (ASBDs). (**A**) Structure of Bet, (**B**) 28-*O*-propynoylbetulin (EB5) and (**C**) 28-*O*-propargyloxycarbonylbetulin (EB25/1).

Two ASBDs with acetylenic ligands at C-28 position were determined according to their anti-cancer potential against pediatric tumor cells in vitro. The chemical structures of the synthesized ASBDs 28-*O*-propynoylbetulin (EB5) and 28-*O*-propargyloxycarbonylbetulin (EB25/1) and their precursor betulin (Bet) are shown in Figure 1A–C.

### 2.1. ASBDs Reduce Viability and Proliferation of Pediatric Cancer Cells Stronger than TMZ and CDDP In Vitro, Whereas They Show Moderate Activity Against Normal Cells

**Figure 2:**
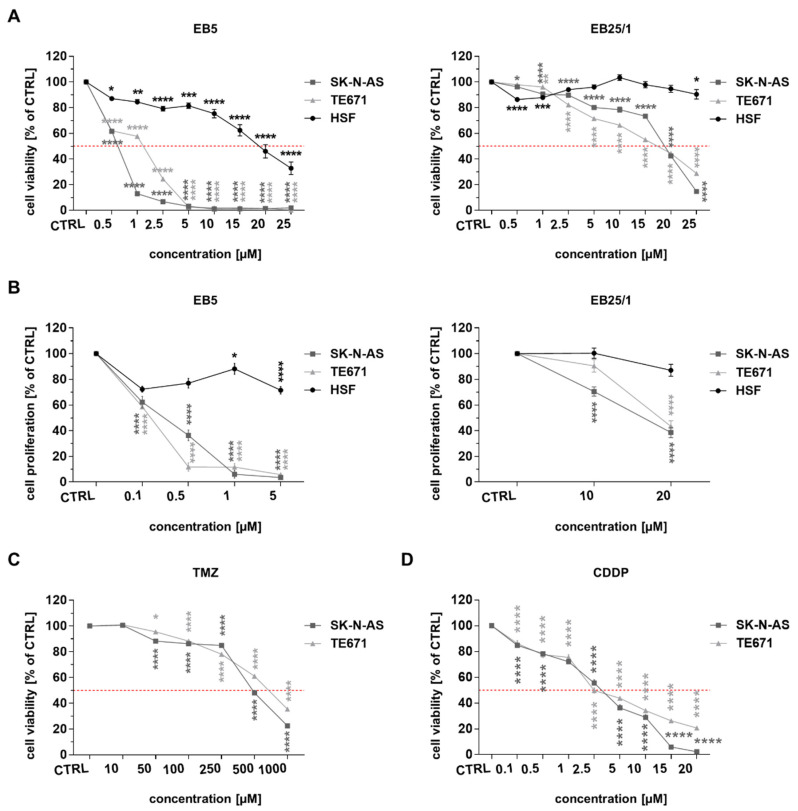
ASBDs reduce viability and proliferation of pediatric cancer cells stronger than TMZ and CDDP in vitro, whereas they show moderate activity against normal cells. SK-N-AS, TE671 and HSF cell lines were treated with ASBDs for 96 h and then analyzed with MTT metabolism assay for cell viability/survival, or for 48 h with BrdU incorporation test for cell proliferation. (**A**) The line graph presents cell viability of SK-N-AS, TE671 and HSF cells after treatment with EB5 or EB25/1. (**B**) The line graph presents cell proliferation of SK-N-AS, TE671 and HSF cells after treatment with EB5 or EB25/1. (**C**) The line graph presents cell viability of SK-N-AS and TE671 cells after treatment with TMZ for 96 h and analyzed with MTT metabolism assay. (**D**) The line graph presents cell viability of SK-N-AS and TE671 cells after treatment with CDDP for 96 h and analyzed with MTT metabolism assay. The results were normalized to control cells (CTRL, treated with 0.1% dimethyl sulfoxide DMSO, as a solvent control) and represent the mean ± SEM of *n* = 32 from 4 independent experiments (MTT assay) or *n* = 24 from 3 independent experiments (BrdU test). Statistical significance was determined by one-way analysis of variance (ANOVA) followed by Dunnett’s post hoc test for multiple comparisons. *p* values were considered significant when *p* ≤ 0.05(*), *p* ≤ 0.01(**), *p* ≤ 0.001(***), *p* ≤ 0.0001(****).

We observed a significant decrease in cell viability (Figure 2A) and proliferation (Figure 2B) in SK-N-AS and TE671 cells after 96 h of treatment with ASBDs in a concentration-dependent manner. HSF cells were much less affected after incubation with ASBDs (Figure 2A,B). When comparing both BE derivatives, EB5 demonstrated a more significantly enhanced effect than EB25/1 against both of the cancer cell lines studied. Interestingly, SK-N-AS cells were more sensitive than TE671 to EB5 treatment, whereas the viability of TE671 cells was affected much more than that of SK-N-AS when exposed to the same concentrations of EB25/1 (Figure 2A). Furthermore, we also estimated the concentrations of ASBDs required for the inhibition of 50% of cell survival (IC_50_, half maximal inhibitory concentration) for each cell line. The results are presented in Table 1.

Noteworthily, the IC_50_ values of EB5 as well as EB25/1 were considerably lower for both pediatric cancer cell lines than the values for HSF cells and than previously demonstrated IC_50_ doses of Bet for SK-N-AS and TE671 cells [18]. In addition, it was not possible to determine the IC_50_ value for EB25/1 within the tested concentrations (0.5–25 µM) for HSF cells (Figure 2A). Moreover, the selectivity index (SI) of EB5 was higher for SKNAS (27.66) than for TE671 (12.80), showing the significant selectivity of this ASBD against these cancer cells. It is worth mentioning that both ASBDs inhibited cancer cell viability/survival at multi-fold lower concentrations (IC_50_ values within the range 0.62–18.77 µM) than TMZ, a chemotherapeutic agent undergoing several clinical trials for potential application in the treatment of patients with relapsed/refractory NB [8,46,47], and RMS [48,49,50], showing the promising clinical relevance of the studied Bet derivatives (Figure 2C and Table 1). CDDP has also been used in the therapy of pediatric cancers, including NB [9,10,11,51] and RMS [52,53,54]. For that reason, we also evaluated the effect of treatment of ASBDs with CDDP on TE671 and SK-N-AS cells’ viability. Both the studied cell lines showed similar and relatively high sensitivity to CDDP (Figure 2D), evidenced by IC_50_ values of 2.48 µM and 3.22 µM for TE671 and SK-N-AS cells, respectively (Table 1).

### 2.2. Combination of ASBDs with CDDP Enhances Cytotoxicity of Both CDDP and EB5 Administered Singly

**Figure 3:**
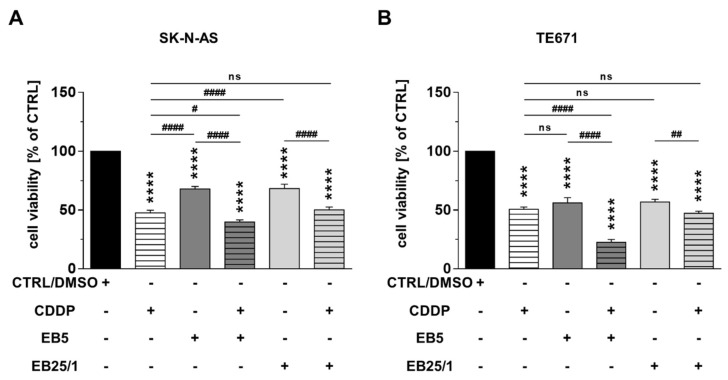
Combination of ASBDs with CDDP enhances cytotoxicity of both CDDP and EB5 administered singly. SK-N-AS and TE671 cell lines were treated with CDDP and ASBDs (the doses of IC_50_ values), and ASBDs in combination with CDDP (the doses of IC_50_ value) for 96 h and then analyzed with MTT metabolism assay for cell viability/survival. (**A**) The bar graph presents cell viability of SK-N-AS cells after treatment with CDDP, EB5, EB25/1 and combination of CDDP with EB5 or EB25/1. (**B**) The bar graph presents cell viability of TE671 cells after treatment with CDDP, EB5, EB25/1 and combination of CDDP with EB5 or EB25/1. The results were normalized to control cells (CTRL, 0.1% DMSO as a solvent control) and represent the mean ± SEM *n* = 32 from 4 independent experiments. Statistical significance was determined by one-way analysis of variance (ANOVA) followed by Tukey’s post hoc test for multiple comparisons. *p* values were considered significant when *p* ≤ 0.0001 (****, vs. CTRL) and *p* ≤ 0.05 (#), *p* ≤ 0.01 (##), *p* ≤ 0.0001 (####).

We also determined the effect of the combined treatment of ASBDs with CDDP on NB and RMS cells’ viability. The mixture of EB5 or EB25/1 and CDDP at concentrations of IC_50_ values decreased the survival rates of SK-N-AS and TE671 cells in a similar way and to a greater extent than the single treatment with ASBDs and, in the case of the EB5 derivative, than single treatment with CDDP. However, no statistically significant augmentation was found after the treatment with the combination of EB25/1 and CDDP in comparison to CDDP administered alone (Figure 3A,B).

### 2.3. ASBDs Inhibit Cell Cycle Progression of Pediatric Cancer Cells by Affecting S Phase

**Figure 4:**
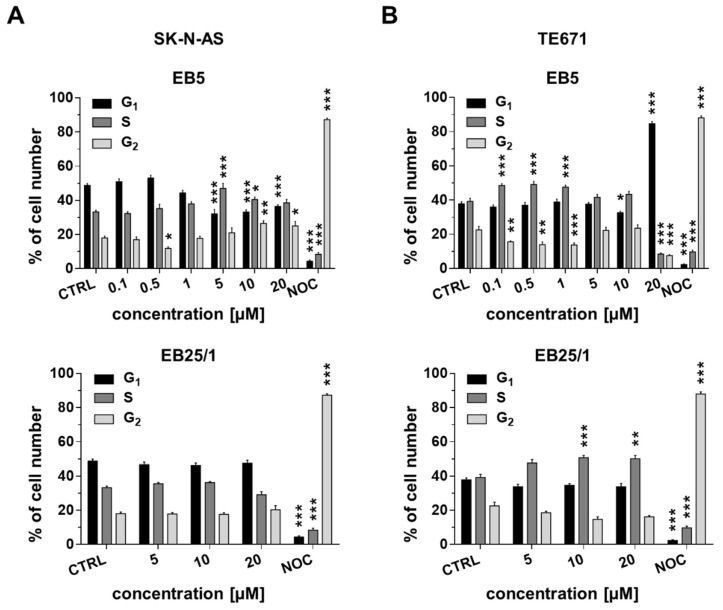
ASBDs inhibit cell cycle progression of pediatric cancer cells by affecting S phase. SK-N-AS and TE671 cell lines were treated with selected concentrations of ASBDs for 24 h, stained with PI, and then analyzed with FACS flow cytometry for cell cycle distribution. (**A**) The bar graphs present percentage of cells in phase G_1_, S and G_2_ in SK-N-AS cells after treatment with EB5 (upper graph) and EB25/1 (bottom graph). (**B**) The bar graphs present percentage of cells in phase G_1_, S and G_2_ in TE671 cells after treatment with EB5 (upper graph) and EB25/1 (bottom graph). Control cells (CTRL) were treated with 0.1% DMSO as a solvent control. Nocodazole (NOC, 5 µg/mL) was used as a reference drug for induction of G_2_/M cell cycle arrest. The results represent the mean ± SEM of *n* = 10 from 5 independent experiments. Statistical significance was determined by two-way analysis of variance (ANOVA) followed by Dunnett’s post hoc test for multiple comparisons. *p* values were considered significant when *p* ≤ 0.05 (*, vs. CTRL), *p* ≤ 0.01 (**, vs. CTRL), *p* ≤ 0.001 (***, vs. CTRL).

We observed an increased percentage of cells in the S phase, followed by a reduction in the number of cells in the G_1_ phase when SK-N-AS cells were exposed to high concentrations of EB5 (5–20 µM). On the other hand, no significant changes in cell cycle progression were found after treatment of the SK-N-AS cell line with EB25/1 (Figure 4A). In TE671 cells, considerable a decrease in cell number in the G_2_ phase accompanied by a prominent increase in the S phase were found after treatment with EB5 (low doses 0.1–1 µM), while the highest concentration (20 µM) induced a growth arrest of cancer cells in the G_1_ phase. In contrast to SK-N-AS cells, after 24 h of treatment with EB25/1, we showed a minor but significant increase in the number of cells in the S phase in TE671 cells (Figure 4B). Altogether, we found a common mechanism mediated by the EB5 derivative in NB and RMS cell lines, resulting in the slowing down of cell cycle progression at S and G_2_ phases.

### 2.4. ASBDs Inhibit Phosphorylation of Kinases Crucial for Growth and Proliferation of Cancer Cells

**Figure 5:**
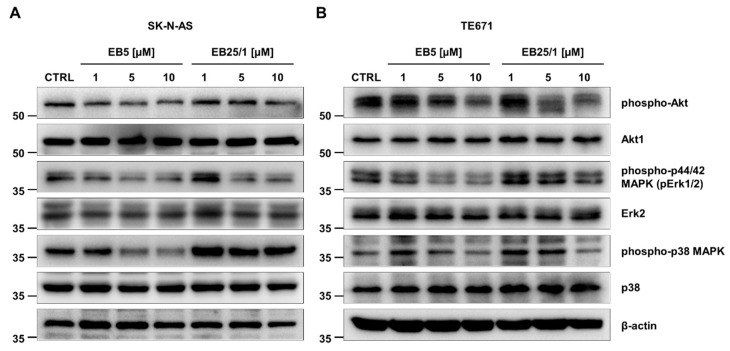
ASBDs inhibit phosphorylation of kinases crucial for growth and proliferation of cancer cells. SK-N-AS and TE671 cell lines were treated with selected concentrations of ASBDs for 3 h and then analyzed with Western blotting for kinases activation. (**A**) Representative immunoblots show the level of phosphorylated Akt and MAP (Erk1/2 and p38) kinases in SK-N-AS cell line after treatment with ASBDs. (**B**) Representative immunoblots show the level of phosphorylated Akt and MAP (Erk1/2 and p38) kinases in TE671 cell line after treatment with ASBDs. Total: Akt1, Erk2, p38 and *β*-actin were used as the loading controls for an equal amount of protein. Control cells (CTRL) were treated with 0.1% DMSO as a solvent control.

To determine the molecular background behind the decrease in pediatric cancer cells’ viability, proliferation and disturbances in cell cycle progression after treatment with ASBDs, we investigated alteration in intracellular signaling pathways essential for the survival and growth of tumor cells, including cascades of PI3K/Akt [55,56] and MAP (Mitogen-Activated Protein) kinases [57,58]. Western blotting analyses demonstrated that the incubation of SK-N-AS cells for 3 h with ASBDs resulted in a reduction in Akt and Erk1/2 kinases’ activity in comparison to control CTRL cells, measured by the phosphorylation and activation status of these kinases. A significant decrease in the phosphorylation of Akt (Ser473), Erk1/2 (Thr202/Tyr204) and p38 (Thr180/Tyr182), and thereby its activity, was observed when SKNAS cells were incubated with EB5, and the inhibition of phosphorylation was more prominent than after treatment with EB25/1 (Figure 5A). Similar inhibitory effects of ASBDs on the level of phospho-Akt, phospoho-Erk1/2 and phospho-p38 kinases when compared to CTRL were found in TE671 cells (Figure 5B).

### 2.5. EB5 Induces Apoptosis of Pediatric Cancer Cells In Vitro in a Concentration- and Time-Dependent Manner

**Figure 6:**
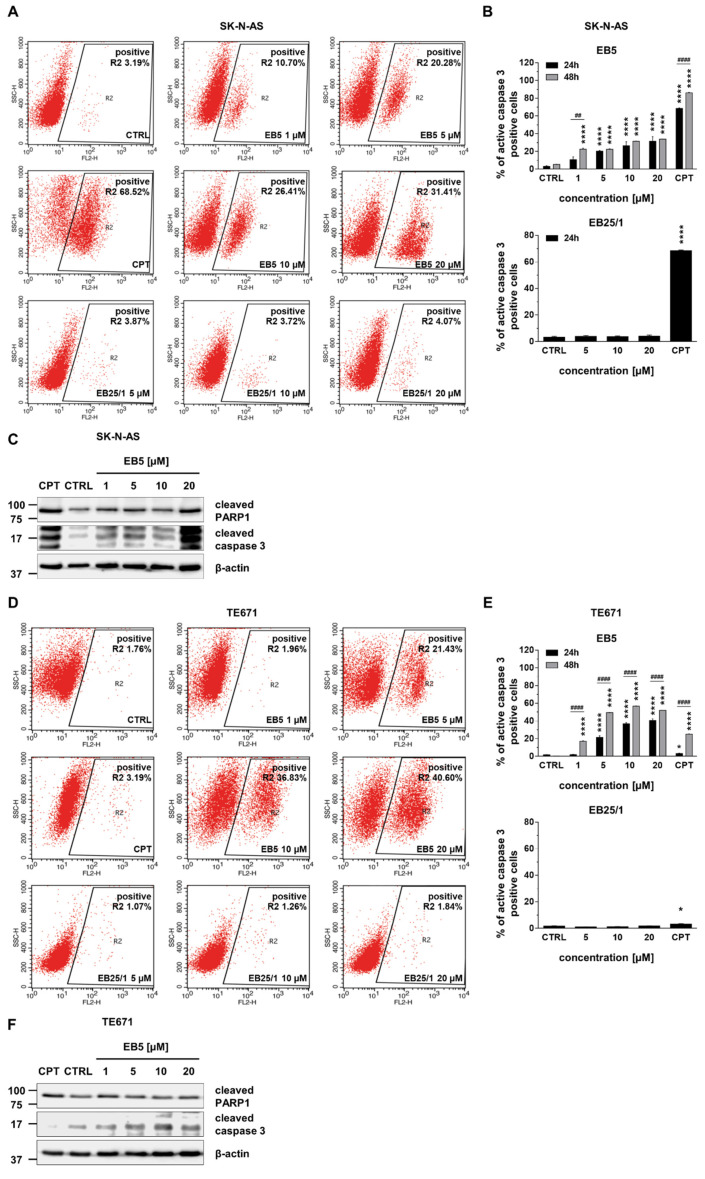
EB5 induces apoptosis of pediatric cancer cells in vitro in a concentration- and time-dependent manner. SK-N-AS and TE671 cell lines were treated with selected concentrations of ASBDs (1–20 µM of EB5, and 5–20 µM of EB25/1) for 24 or 48 h and then analyzed with FACS flow cytometry and Western blotting for induction of apoptotic cell death. (**A**) Representative dot plots show gating of cell subpopulation with activated caspase 3 in SK-N-AS cells. (**B**) The bar graphs present quantification of caspase 3-positive cells percentage in SK-N-AS cell line after treatment with EB5 (for 24 h and 48 h, upper graph) and EB25/1 (for 24 h, bottom graph). The results represent the mean ± SEM of *n* = 6 from 3 independent experiments. Statistical significance was determined by one-way analysis of variance (ANOVA) followed by Dunnett’s post hoc test for multiple comparisons (bottom graph) or two-way ANOVA followed by Sidak’s post hoc test for multiple comparisons (upper graph 24 h vs. 48 h). *p* values were considered significant when *p* ≤ 0.0001 (****, vs. CTRL), and *p* ≤ 0.01 (##), *p* ≤ 0.0001 (####). (**C**) Representative immunoblots show the level of cell death markers (cleaved PARP1 and cleaved caspase 3) in SK-N-AS cell line. (**D**) Representative dot plots show the gating of cell subpopulation with activated caspase 3 in TE671 cells. (**E**) The bar graphs present the quantification of caspase 3-positive cell percentage in TE671 cell line after treatment with EB5 (for 24 h and 48 h, upper graph) and EB25/1 (for 24 h, bottom graph). The results represent the mean ± SEM of *n* = 6 from 3 independent experiments. Statistical significance was determined by one-way analysis of variance (ANOVA) followed by Dunnett’s post hoc test for multiple comparisons (bottom graph) and two-way ANOVA followed by Sidak’s post hoc test for multiple comparisons (upper graph 24 h vs. 48 h). *p* values were considered significant when *p* ≤ 0.05 (*, vs. CTRL), *p* ≤ 0.0001 (****, vs. CTRL), and *p* ≤ 0.0001 (####). (**F**) Representative immunoblots show the level of cell death markers (cleaved PARP1 and cleaved caspase 3) in TE671 cell line. *β*-actin was used as a loading control for an equal amount of protein. Control cells (CTRL) were treated with 0.1% DMSO as a solvent control. Camptothecin (CPT, 20 µM) was used as a reference drug for induction of apoptosis.

Further, we investigated whether cytotoxic and anti-proliferative effects of ASBDs in tumor cells were due to the activation of apoptotic cell death. Therefore, the activation of caspase 3 in cancer cells was assessed using FACS flow cytometry. We observed no statistically significant induction of caspase 3 in both cell lines after 24 h of treatment with EB25/1. On the contrary, the prominent activation of caspase 3 was detected in SK-N-AS and TE671 cells exposed to EB5, and the effect was concentration-dependent (Figure 6A,B,D,E). What is more, in TE671 cells, the pro-apoptotic activity of EB5 was time-dependent. When the incubation time of treatment with EB5 was extended to 48 h, we observed a considerable increase in activated caspase 3-positive cell percentage in comparison to 24 h (Figure 6E), while in SK-N-AS cells, a similar effect was only found at the lowest concentration (1 µM) (Figure 6B). In addition, we confirmed the induction of apoptosis after 24 h of treatment with selected concentrations of EB5 derivative (1–20 µM) by immunodetection of apoptotic cell death markers using Western blotting (Figure 6C,F). The exposure of both SK-N-AS and TE671 cells to EB5 showed the activation of PARP1 and the cleavage of caspase 3. The effects were concentration-dependent—the strongest activation was shown at the highest used dose (20 µM) (Figure 6A,F).

### 2.6. In Silico Study of Physicochemical Parameters, Pharmacokinetic Profile and Druglikeness of ASBDs

To gain a deeper insight into mechanisms of action of the analyzed Bet derivatives, we calculated several properties pivotal for biological and pharmacological activity of chemical compounds, contributing to their usefulness in medicinal chemistry with prospective relevance for future application in clinical practice [59]. The most important physicochemical parameters, including molecular weight (MW), the number of hydrogen bond acceptors (H-BA) and donors (H-BD) and topological polar surface area (tPSA) have been previously determined for Bet and both ASBDs using an in silico approach [60,61]. Here, we estimated lipophilicity (n-octanol/water partition coefficient, LogP) and water solubility (LogS) by different computational methods, followed by comparison of theoretically calculated values of LogP with the experimentally data formerly obtained by reversed-phase thin layer chromatography (RP-TLC) [61]. The results are shown in Table 2.

By using in silico approach, we found the increased lipophilicity of both ASBDs, as their values of LogP calculated with different methods were higher when compared to Bet in the rank order Bet < EB5 < EB25/1, excluding MLOGP and SILICOS-IT algorithms, which indicated the increase in LogP in the rank order Bet < EB25/1 < EB5. For Bet, the values of LogP computed with iLOGP and MLOGP were the closest to the values of LogP_TLC_ obtained experimentally, whereas for both ASBDs, the values calculated by the WLOGP and SILICOS-IT methods were the most similar to LogP_TLC_. Following this, a slight decrease in water solubility (expressed as LogS) in the rank order Bet < EB5 < EB25/1 was determined by using ESOL, Ali and SILICOS-IT algorithms.

For the prediction of ASBDs’ drug-like natures and therefore potential usefulness in medicinal chemistry, we determined the relationship between several physicochemical parameters and pharmacokinetic profile in silico by evaluating the compliance with the five different most popular druglikeness predictive models. The results are shown in Table 3.

Derivative EB5, similarly to Bet, only violated the Lipinski druglikeness guidelines once (MLOGP > 4.15), whereas two violations by EB25/1 were found (MW > 500, MLOGP > 4.15). The Ghose, Egan and Muegge models were violated ≥ once by Bet, as well as both ASBDs and did not meet these druglikeness rules. Following this, the computational predictions based on the Lipinski “rule-of-five” included Bet and EB5, in contrast to EB25/1, within the range of acceptable bioavailability score (0.17—failed, whereas 0.55—passed the Lipinski “rule-of-five”). All the analyzed compounds followed the Veber guidelines with no violations.

Further, using in silico approaches, we predicted the pharmacokinetic profile, ADMET (Absorption, Distribution, Metabolism, Excretion and Toxicity), of the studied Bet derivatives, which may significantly affect the probability of ASBDs being developed as drug candidates in humans. The results are presented in Table 4.

Considering absorption, ADMET analysis determined both EB5 and EB25/1 to be compounds with good HIA; however, a lack of Caco-2-cell permeation and no human oral bioavailability were predicted. In terms of cell and tissue distribution, computational algorithms assessed the ability for permeation across the BBB (blood–brain barrier) by ASBDs, in contrast to Bet. Moreover, different localizations within the cell was predicted—Bet were predicted to be localized in lysosomes, whereas both ASBDs in mitochondria. Bet and ASBDs also expressed strong plasma protein binding activity and were shown as both non-inhibitors and non-substrates for P-glycoprotein. In silico analysis of metabolism revealed an EB25/1 derivative as a substrate for CYP450 isoforms: 2C9 and 3A4, and Bet for CYP450 3A4, whereas none of the analyzed compounds were determined as inhibitors of any of CYP450 isoforms. Through computational estimation of inhibitory effects on drug transporters, we found that ASBDs as well as Bet may potentially be inhibitors of BSEP and OATP isoforms 1B1 and 1B3; however, no inhibitory effect was predicted on OCT2 and MATE1 transporting proteins. Properties analyzed within the ADMET profile also included organ and genomic toxicity. No mutagenic and carcinogenic properties of ASBDs were predicted. However, EB25/1 may potentially induce hepatotoxicity and eye corrosion. In silico evaluation classified Bet and EB5 as compounds belonging to category III of acute oral toxicity, with the values of 3.348 and 3.355 mol/kg, respectively.

Furthermore, potential therapeutic target proteins for analyzed ASBDs were also predicted in silico. The results are presented in Table 5. Interestingly, for Bet and both ASBDs, several proteins belonging mainly to the G protein coupled receptor 1 (including cannabinoid receptor, G protein coupled bile acid receptor 1, delta-, kappa- and mu-type opioid receptors and Epstein–Barr virus-induced molecule 2) and nuclear hormone receptor families (including glucocorticoid receptor and estrogen receptor-*α*) were found among the 10 top-ranked potential targets (with the highest predictive scores). Following the predicted target proteins discriminated EB5 and EB25/1 from Bet: metabotropic glutamate receptor 5 (mGluR5), 3-hydroxy-3-methylglutaryl-coenzyme A reductase (HMG-CoA reductase), nuclear factor erythroid 2-related factor 2 (NRF2) and muscarinic acetylcholine receptor M3 (mAChR M3).

Subsequently, the signal transduction pathways potentially targeted by Bet and both analyzed ASBDs were also calculated using the computational method. The results are shown in Table 6. Among the 10 top-ranked pathways (with the highest predictive scores), we predicted several signaling pathways common for Bet as well as both ASBDs. These signal transduction networks, including complement and coagulation cascades, focal adhesion, axis PI3K-Akt, the hormone-related pathway, ferroptosis, mineral and protein absorption and p53 signaling, play important roles in a variety of cellular processes crucial for cell growth, death, differentiation and carcinogenesis. More importantly, the computational prediction of the PI3K-Akt signaling pathway as a target of ASBDs is consistent with our findings from Western blot analysis.

## 3. Discussion

Significant advancements in the understanding of the genetic and molecular landscape of the most prevalent pediatric tumors in recent years, following the identification of new potential therapeutic targets [85,86,87], create new possibilities for treatment. However, no significant progress has been observed in the survival of patients with advanced or metastatic disease, and therapeutic protocols for the treatment of NR and RMS have not changed prominently since the 1980s [4,88,89,90]. Currently available treatment modalities, including surgical resection, radiation and standard chemotherapy, are ineffective and related to numerous heavy adverse effects. Subsequently, pediatric cancers still remain a significant clinical challenge [5,7,14]. Therefore, multi-agent treatment and/or a multi-modality approach is urgently required.

Plant-derived chemicals are widespread in nature and are thus relatively readily available. Many of them have been proved to show a large spectrum of biological and pharmacological activities, including anti-bacterial, anti-viral, anti-fungal, anti-parasitic, anti-inflammatory [91,92], anti-oxidant and chemopreventive properties [91,92,93,94,95,96]. Consequently, significantly increasing interest in the use of natural substances as potential agents in the prevention and treatment of many human diseases, including cancer, is being observed nowadays [97,98,99,100]. Following this, the modification of phytochemicals with anti-tumor properties verified in cellular and animal models is a well-known method to obtain new compounds with improved pharmacokinetic parameters, activity and selectivity towards cancer cells for potential implementation in oncological treatment [99,101,102].

Bet isolated from birch bark has been shown to demonstrate interesting chemopreventive and anti-cancer activities in vitro and in vivo, as previously reviewed in great detail [16,17]. The structure of Bet molecules makes several chemical modifications possible and thus it has been used as a starting compound for the synthesis of numerous new derivatives with potential applications in chemotherapy. The synthesis of mono- and di-acetylenic derivatives of Bet bearing acetyl substituents at the position of C-28 was reported for the first time in 2010 [103]. In the present research, we showed the promising anti-cancer potential of two ASBDs carrying an acetylenic side chain at carbon C-28: 28-*O*-propynoylbetulin (EB5) and 28-*O*-propargyloxycarbonylbetulin (EB25/1) towards pediatric cancers cells in vitro. We found that both ASBDs prominently reduced cancer cells’ viability/survival and proliferation in a dose-dependent manner, also showing high selectivity for tumor cells measured by the selectivity index and relatively low or moderate activity against normal cells. More importantly, here, we demonstrated the considerably enhanced anti-survival and cytotoxic potential of ASBDs in comparison to IC_50_ values of precursor compound Bet in SK-N-AS and TE671 cell lines showed previously [18]. EB5, a derivative of Bet with a shorter alkynyl chain, showed evident stronger anti-survival, cytotoxic and anti-proliferative properties towards the analyzed pediatric cancer cells than EB25/1, a derivative carrying a longer alkynyl chain. The results from our in vitro study were highly consistent with the structure–activity relationships (SARs) in the literature, showing that the biological and pharmacological activity of ASBDs, including anti-tumor properties, was determined by differences in their chemical structure, especially their substituents. Several previous reports have showed that the introduction of an acetylenic side chain into the Bet structure resulted in the prominently increased anti-cancer activity of the derivatives when compared to a precursor molecule [18,25,60,61,103,104]. The carbon–carbon triple bond is considered to be one of the most crucial functional groups in organic and medicinal chemistry. The incorporation of alkyne moiety (acetylenic side chain, such as propynoyl in EB5) which contain a C-C triple bond was found essential for chemical, physical and biological properties of the molecule which determine its pharmacological profile. This simple modification at the position C-28 adjacent to the carbonyl group leads to formation of a very reactive chemical structure which may easily interact with other ligands and molecules [104,105,106]. Therefore, alkyne substituents may enhance the affinity of the modified molecule to the nucleophilic amine and/or thiol groups in proteins within the cell membranes, following disturbances in their stability and integrity, and finally cause a decrease in cell viability and survival. Consequently, it is considered that alkyne groups at C-28 augment the affinity to the nucleophilic amine or thiol groups of proteins in the cellular membranes, affecting their integrity and stability and thus decreasing cell viability and survival [60,104,106]. Similar to our study, other reports demonstrated the potent cytotoxicity and anti-proliferative activity of ASBDs towards cancer cells in vitro, including melanoma (G-361 cell line) [104], human leukemia (CCRF/CEM) and murine leukemia (P388) [25]. We have previously demonstrated that EB5 and EB25/1 significantly decreased the cell survival and proliferation of glioma cells (T98G and C6 cell lines), with IC_50_ values being several-fold lower than those of TMZ when tested in vitro [107]. In this study, we also showed several times (6.8 to 26) lower IC_50_ values of EB5 for pediatric cancer cell lines than those observed for glioma cells, while the IC_50_ doses of EB25/1 were quite similar in these different cellular models [107]. Additionally, here, we found that EB5 showed increased cytotoxicity compared to conventional chemotherapeutic drug, CDDP, in analyzed cancer cell lines, and the combination of EB5 with CDDP at concentrations of IC_50_ values considerably augmented anti-cancer activity in comparison to single treatment with EB5 or CDDP. Another study also showed that 28-acetylenic derivatives of Bet showed noteworthy anti-tumor effects towards human cancers of head and neck, ovarian, colon, lung, breast, thyroid and liposarcoma [103]. Given the fact that the analyzed ASBDs demonstrated relatively weak cytotoxicity against human normal cells within the tested concentrations (0.5–25 µM) and the concentrations required to exert significant effects on viability/survival were multi-fold lower than these of TMZ, a chemotherapeutic drug considered for application in the therapy of NB [8,46,47] and RMS [48,49,50] patients, our research creates the opportunity for the development of new promising anti-tumor agents. Moreover, as CDDP is commonly used for the therapy of NB [9,10,11,51] and soft tissue malignancies, including RMS [52,53,54], the combination of CDDP/EB5 supports the rationale for the usefulness of Bet derivatives combined with CDDP in the future development of novel therapeutic schedules. This kind of approach may potentially overcome frequently observed drug resistance and could result in the enhancement of anti-cancer activity and the reduction in toxicity and adverse effects [10,108] by a significant decrease in CDDP doses in clinical use. However, the pharmacological type of interactions between ASBDs and CDDP requires further analysis.

Next, we investigated the molecular mechanism underlying the ASBD-driven inhibition of pediatric cancer cells’ growth and proliferation. EB5 and other 28-acetylenic derivatives were also previously demonstrated as pro-apoptotic agents in ovarian A2780 and colon cancer SW480 [103] and melanoma G-361 [104] cell lines. We demonstrated the dose-dependent induction of apoptosis evidenced as upregulated levels of cleaved PARP and cleaved caspase 3, followed by the inhibition of signal transduction pathways commonly hyper-activated in many human tumors: Akt [55,56] and MAP kinases (Erk1/2 and p38) [57,58]. Involvement of the Akt pathway in the ASBD-mediated mechanism of action was also suggested by in silico studies. However, we cannot exclude other signaling pathways that may potentially contribute to anti-survival and anti-proliferative effects of ASBDs in analyzed cancer cell lines.

A growing number of evidence suggests that the early in silico evaluation of the ADMET profile and prediction of druglikeness of potential drug candidates play important roles in contemporary medicinal chemistry. Since the discovery and development of novel effective medicines and their subsequent implementation into clinical practice are extremely time- and cost-consuming processes, preliminary computational analysis would help in a significant decrease in failures in the clinical phases following an undesirable pharmacokinetic profile or unacceptable toxicity [109,110,111]. Therefore, in this study, we also estimated the pharmacokinetic parameters and drug-like properties of ASBDs by the state-of-the-art in silico approach, implementing machine learning methods and algorithms.

Bet and its natural and synthetic derivatives, as many plant-derived secondary metabolites, are frequently insoluble or show very poor aqueous solubility [31,103,112,113]. Relatively low values of LogP determined for Bet are a consequence of two hydroxyl groups within its molecule, whereas the introduction of an acetyl moiety bearing additional carbon atoms into the Bet structure was followed by an increase in lipophilicity of ASBDs [61]. In silico predictions, independently of the computational method used, revealed the considerable increase in LogP accompanied by diminished hydrosolubility (LogS) in the rank order Bet < EB5 < EB25/1. These results obtained by theoretical calculations are in agreement with previous experimental data from RT-TLC [61]. Although the increasing lipophilicity leads to high fat solubility and consequently, improved lipid membranes permeation, the subsequent lowering of solubility in water may create several problems for the pharmacokinetic properties of the drugs, such as decreased absorption and distribution, and thus may result in limited in vivo administration and low bioavailability [114,115]. Nevertheless, this kind of obstacle could potentially be overcome by the complexation of ASBDs with hydrophilic carriers, such as *β*-cyclodextrin [113] and *γ*-cyclodextrin derivatives [31], by incorporation in nanoemulsion [34] or encapsulation into hydrophilic vehicles, including liposomes [34,103]. On the other hand, our computational analysis showed the potential ability of ASBDs to pass through the BBB, since increasing lipophilicity may often significantly improve BBB permeation according to some literature reports [116,117]. Noteworthily, since we previously found significant anti-cancer effects of both EB5 and EB25/1 in glioma cells in vitro [107], these properties of ASBDs may have potentially clinical-related relevance in the future if they are confirmed in a preclinical in vivo model. Our in silico analysis of ADMET parameters estimated good HIA of Bet and ASBDs; however, no permeability in the Caco-2 cell model and a lack of human oral bioavailability were predicted. In contrast, previously determined tPSA values for Bet and its acetylenic derivatives [60] were less than 140, which may potentially suggest high oral bioavailability [118,119]. Several physicochemical parameters based on chemical structure, including molecular weight, lipophilicity, the number of H-BA and H-BD, tPSA, the number of atoms and rotatable bonds, were required for the evaluation of the probability of ASBDs being developed as medicines for humans. A useful method to predict a drug-like properties of the drug candidate is to determine its compliance with some druglikeness predictive guidelines. Bet and its derivative, EB5, were computationally demonstrated to follow two out of five drug-likeness guidelines: Lipinski and Veber, whereas for EB25/1, only Veber. According to the Lipinski “rule-of-five”, which is the most commonly used, and based on the observations that most orally administered medicines are rather small and moderately lipophilic molecules, an orally active drug candidate should not violate more than one of the following rules: MW less than 500 g/mol, MLOGP ≤ 4.15 (or LogP ≤ 5), number of H-bond acceptors (N or O atoms) less than 10, number of H-bond donors (NH or OH groups) ≤ 5 [67,120]. Here, we determined that Bet and derivative EB5 would fulfill the Lipinski “rule-of-five” with only one violation related to lipophilicity, while EB25/1 was found to violate two of these parameters since its MW is greater than 500 g/mol, and MLOGP ≥ 4.15 (LogP ≥ 5), and potentially, poor absorption or permeation is more likely for this Bet derivative, resulting in a higher probability of low human oral bioavailability. On the other hand, Bet and both ASBDs fulfilled the Veber druglikeness predictive guideline, which is only based on the number of rotatable bonds (≤10) and tPSA value (≤140) [72]. Interestingly, other reports have shown that numerous well-known natural-derived medicinal agents, including macrocycles and cyclic peptides, may become human drugs in spite of the fact that they violate the Lipinski “rule-of-five” [121,122,123].

In contrast to Bet’s subcellular localization of EB5 and EB25/1 predicted by ADMET, computational analysis may suggest potential diverse distribution and metabolic pathways between both ASBDs and their precursor molecule. The studied Bet derivatives, based on physicochemical properties, were demonstrated as neither substrates nor inhibitors of P-glycoprotein (P-gp) and subsequently could be prevented from undesirable efflux outside the cells, which is commonly involved in the multidrug resistance of numerous cancers to standard chemotherapeutics. P-gp, a transmembrane protein, is responsible for the transport of xenobiotics, including drugs out of the cells, and therefore can play a crucial role in the absorption and distribution of medicines [124,125]. Other protein families important for the transport and disposition of drugs are solute carrier (SLC) transporters: organic anion transporting polypeptides (OATPs), multidrug and toxin extrusion (MATE) and organic cation transporters (OCTs), which may affect the intracellular levels of several medications. OATPs are involved in the influx of endogenous and exogenous substances and mediate the hepatic uptake and distribution of endogenous agents and commonly used medicines, including standard chemotherapeutics [111,126,127], whereas OCTs are poly-specific membrane transporters in the liver and kidney which contribute to the hepatic uptake of small, hydrophilic, positively charged molecules [111,128,129,130]. In contrast, MATEs have been described as proton/cation antiporters within plasma membrane and play important roles mainly in the efflux of xenobiotics, including the renal clearance of clinically used drugs [128,129,130]. Our computational analysis of the ADMET profile predicted inhibitory effects of both Bet and ASBDs on OATP 1B1 and 1B3 isoforms, thus indicating their potential emerging role as candidates for pharmacological inhibitors of OATPs, since these transporting proteins were found to be significantly overexpressed in numerous human tumors suggesting the possible function of OATPs in cancerogenesis [127]. Conversely, Bet and both EB5 and EB25/1 were determined as non-inhibitors of OCT2 and MATE1 transporters that could possibly suggest no ability to block OCT-mediated transport. Computational analyses have also found a potential inhibitory effect of Bet and ASBDs on the bile salt export pump (BSEP), suggesting possible involvement in liver and bile homeostasis, which could result in the increased concentration of bile salt in serum since BSEPs are the main transporters responsible for the secretion of bile acids and salts from hepatic cells [131,132].

The metabolism of chemical compounds has been regarded as one of the most crucial and difficult to predict parameters that should be taken into consideration during the design and development of novel drug candidates and ought to be carefully monitored in preclinical and clinical validations [111,133]. The cytochrome CYPP450 superfamily is a well-described large group of enzymes involved in the metabolic transformation of both endogenous and exogenous compounds [134], including anti-cancer drugs or toxins, mainly in phase I reactions of oxidative biotransformations of many xenobiotics [135]. Although, in silico analysis found ASBDs as non-inhibitors of most CYP450 isoforms, derivative EB25/1 was predicted as a substrate for CYP450 3A4 and CYP450 2C9, whereas Bet was predicted for CYP450 3A4. Essentially, neither EB5 nor EB25/1 were found to be carcinogenic/mutagenic or highly toxic agents, and they were classified as belonging to category III of toxicity according to the EPA [78] (slightly toxic/irritating); however, EB25/1 was predicted as potentially hepatotoxic and corrosive to the eye.

The in silico prediction of potential biological targets within the cell or tissue for the analyzed ASBDs may also provide an insight into the molecular mechanisms underlying their anti-cancer activity. In this study, the proteins and signaling pathways which could be therapeutic targets for Bet derivatives were determined with the Balanced Substructure–Drug–Target Network-Based Inference (bSDTNBI) method using the NetInfer web server [79,80,81]. Interestingly, among 10 targets of Bet as well as ASBDs with the highest scores, we found several members of the G protein-coupled receptor 1 (GPCRs) family and nuclear hormone receptor family. GPCRs are transmembrane proteins involved in the transduction of extracellular signals into cells, following the activation of G proteins and the modulation of numerous physiological and pathological processes, including tumorigenesis [136,137]. Nuclear hormone receptors are specialized transcription factors responsible for the regulation of specific gene expression by the promotion or repression of transcription [138,139]. Noteworthily, both these protein families are considered as the major targets for potential pharmacological intervention in several human diseases [140,141], which may suggest the future clinical relevance of the studied ASBDs. Furthermore, some target signaling cascades such as focal adhesion, PI3K-Akt, PPAR, ferroptosis and the p53 protein pathway predicted for both EB5 and EB25/1 in silico were found to be directly related to development and progression of human cancers. The targeting of focal adhesion proteins has been demonstrated to overcome the resistance of tumor cells to standard treatment modalities, such as chemo- and radiotherapy [142,143]. PI3K-Akt signaling is well known for the promotion of cancer survival, growth and proliferation, and its several pharmacological inhibitors are currently undergoing clinical trials or have been recently implemented into the clinical practice [144,145]. Importantly, computational prediction of the PI3K-Akt axis as one of the signal transduction pathways potentially affected by ASBDs was highly consistent with our findings from Western blot analysis, which showed reduced levels of phosphorylated Akt, following its decreased activity after treatment with both EB5 and EB25/1. The peroxisome proliferator-activated receptors (PPARs) are members of the ligand-inducible nuclear hormone receptor family involved in several processes related to metabolism and inflammation [146,147]. The regulation of the PPAR signaling cascade might be a possible new strategy to prevent carcinogenesis and progression [148,149]. Another signaling pathway potentially targeted by ASBDs was ferroptosis, a novel, recently discovered form of programmed cell death considered to be an interesting and promising player in the therapy of cancers [150,151]. Other pathways predicted as molecular targets for ASBDs which have not been directly linked to cancer so far, including complement and coagulation cascades, estrogen signaling, protein digestion and absorption, mineral absorption or hematopoietic cell lineage, need further investigation and confirmation in vitro and in vivo. Though it is difficult to speculate about the potential effects of ASBDs on the aforementioned signal transduction pathways based only on computational calculations, nevertheless, these findings may determine the directions for future molecular studies of both EB5 and EB25/1 and suggest new aspects to be analyzed in great detail.

## 4. Material and Methods

### 4.1. Synthesis of ASBDs

**Figure 7:**
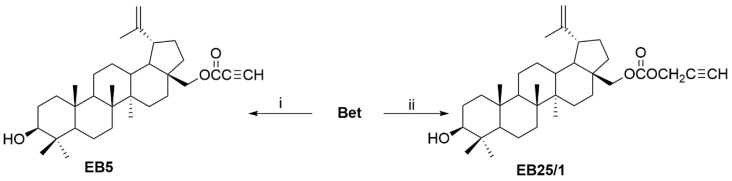
Synthesis of ASBDs. Reagents and reaction conditions: i—propynoic acid, DDC (N,N′-dicyclohexylcarbodiimide), DMAP (4-dimethylaminopyridine), dichloromethane, from −10 °C to room temperature; ii—propargyl chloroformate, pyridine, benzene, from −5 °C to room temperature [25].

The ASBDs (Figure 1) were synthesized in the Department of Organic Chemistry at the Medical University of Silesia in Sosnowiec, as previously described [25]. The reaction of Bet with propynoic acid was carried by the Steglich method to give 28-*O*-propynoylbetulin (EB5). Bet was converted to the 28-*O*-propargyloxycarbonylbetulin (EB25/1) by esterification with propargyl chloroformate with the presence of pyridine in benzene (Figure 7. Both ASBDs were purified using gel column chromatography. The structures of ASBDs were determined on the basis of their ^1^H- and ^13^C-NMR (Bruker AVANCE III HD 600, Billerica, MA, USA, deuterated chloroform), IR (IRAffinity-1 FTIR spectrometer; Shimadzu Corporation, Kyoto, Japan, KBr pellet), and MS spectra (Bruker Impact II, Billerica, MA, USA). The ^1^H- and ^13^C-NMR spectral data for ASBDs were consistent with the literature values (Supplementary Materials, Figures S1–S4).

### 4.2. Cell Culture and Treatment

Human neuroblastoma SK-N-AS and human rhabdomyosarcoma TE671 cell lines were purchased from the American Type Culture Collection (ATCC, Manassas, Virginia, USA) and the European Collection of Authenticated Cell Cultures (ECACC, Salisbury, UK), respectively. Normal human primary fibroblast culture (HSF) was obtained via the out-growth technique from skin explants of a young person, using a method routinely ongoing in our lab (Local Ethical Committee permission No KE-0254/298/2015, approved on 26 November 2015) and cultured as previously described [152]. Pediatric cancer cells were cultured in Dulbecco’s Modified Eagle’s Medium/Nutrient F-12 Ham (DMEM/F12, Sigma-Aldrich, St. Louis, MO, USA) supplemented with 10% fetal bovine serum (FBS, Sigma-Aldrich, St. Louis, MO, USA) and antibiotics: 100 U/mL of penicillin and 100 µg/mL of streptomycin (Sigma-Aldrich, St. Louis, MO, USA). The cell cultures were regularly tested for contamination with *Mycoplasma* sp. The medium was replaced frequently at 3-day intervals. Sub-confluent cells were rinsed with a phosphate-buffered solution (PBS, Biomed Lublin, Poland) without Ca^2+^/Mg^2+^ and harvested with 0.25% Trypsin-EDTA (Sigma-Aldrich, St. Louis, MO, USA). The cell cultures were maintained in a humidified atmosphere of 5% CO_2_ and 95% air, at 37 °C, as previously described [153].

ASBDs were dissolved in DMSO (Sigma-Aldrich, St. Louis, MO, USA) to prepare fresh stock solution before each experiment (25 mM for EB5 and 50 mM for EB25/1). CDDP (Sigma-Aldrich, Munich, Germany) was dissolved in PBS as a 10 mM stock solution. TMZ (Sigma-Aldrich, Munich, Germany) was dissolved in DMSO as a 150 mM stock solution. To exclude additional toxic effects of DMSO, the solvent was used as a control. For treatment, all solutions were prepared in fresh, complete culture medium.

### 4.3. MTT Metabolism Assay

Cell viability and survival was determined by measuring the activity of mitochondrial dehydrogenase to reduce a yellow tetrazolium salt MTT (3-[4,5-dimethylthiazol-2-yl]-2,5-diphenyltetrazolium bromide) to its purple formazan (insoluble crystals), as previously described [154]. As MTT is reduced in the mitochondria of metabolically active cells, the amount of formazan is proportional to the number of living cells. Briefly, SK-N-AS and TE671 cells were seeded on 96-well microplates at a density of 5.0 × 10^3^ and 1.0 × 10^3^ cells per well, respectively, and left overnight to attach. Then, the culture medium was removed, and cells were exposed to increasing concentrations of ASBDs (0.5–25 µM) or DMSO (CTRL, solvent control). Cell viability was assessed after 96 h, followed by incubation for 3 h with MTT solution (final concentration of 0.5 mg/mL in PBS). Then, formazan crystals were solubilized overnight in lysis buffer (10% SDS in 0.01 M HCl). Finally, the absorbance at 570 nm wavelength was measured using Microplate Reader TECAN Infinite M200 Pro (Tecan Group Ltd., Männedorf, Switzerland) operating with Microplate Manager version 5.0 (Bio-Rad Laboratories, Hercules, CA, USA).

### 4.4. Calculation of Half Maximal Inhibitory Concentration and Selectivity Index

The half maximal Inhibitory Concentration (IC_50_) is a concentration inhibiting the growth and survival of 50% of the treated cells in comparison to untreated control cells. IC_50_ values were calculated via linear regression analysis using the dose–response curves from the MTT metabolism assay. The selectivity index (SI) was calculated as a ratio of IC_50_ for human normal cells HSF and IC_50_ for each cancer cell line.

### 4.5. BrdU Incorporation Test

Cell proliferation was determined using colorimetric immunoassay based on the measurement of bromodeoxyuridine (BrdU), a thymidine analogue incorporated into DNA strands during the synthesis of DNA, as previously described [155]. The reaction product was quantified by measuring the absorbance which directly correlates to the amount of newly synthesized DNA and to the number of proliferating cells. In brief, SK-N-AS and TE671 cells were seeded on 96-well microplates at a density of 3.0 × 10^3^ and 1.0 × 10^3^ cells per well, respectively, and left overnight to attach. Then, culture medium was removed, and cells were exposed to increasing concentrations of ASBDs (0.5–20 µM) or DMSO. Cell proliferation was assessed after 48 h. The commercially available Cell Proliferation ELISA BrdU Kit (Roche Diagnostics GmbH, Mannheim, Germany) was used in accordance with the manufacturer’s protocols. Finally, the absorbance at a wavelength of 450 nm was measured using Microplate Reader TECAN Infinite M200 Pro (Tecan Group Ltd., Männedorf, Switzerland) operating with Microplate Manager 5.0 software (Bio-Rad Laboratories, Hercules, CA, USA).

### 4.6. Cell Cycle Analysis by FACS Flow Cytometry

Cell cycle progression was investigated via the measurement of propidium iodide (PI) incorporated into DNA strands by FACS flow cytometry. Briefly, SK-N-AS and TE671 cells were seeded on 6-well plates at a density of 1.0 × 10^6^ and 0.5 × 10^6^ cells per well, respectively, and left overnight to attach. Then, the culture medium was removed, and the cells were exposed to selected concentrations of ASBDs or DMSO. After 24 h of treatment, tumor cells were harvested using accutase solution, then centrifuged at 300× *g* for 5 min at RT, and the pellets were fixed in ice-cold 80% ethanol overnight at −20°C. Next, the cells were centrifuged at 300× *g* for 5 min at +4 °C, washed twice with PBS and labelled with 0.5 mL of PI/RNase Staining Buffer (BD Pharmingen™, BD Biosciences, San Diego, CA, USA) per 1 × 10^6^ cells for 15 min at RT, in the darkness. Then, the cells were analyzed using a flow cytometer FACSCalibur (Becton Dickinson, San Jose, CA, USA). Nocodazole (5 µg/mL) was used as a reference drug inducing G_2_/M cell cycle arrest, showing the functionality of the procedure. The PI fluorescence intensities of individual nuclei were measured using the excitation wavelength at 488 nm (an argon laser) and 560 nm dichromatic mirror and 600 nm band pass filter. Cell debris were excluded from analysis by appropriate light scatter gating. At least 10,000 events for each sample were acquired, and the data were analyzed using CellQuest software (Becton Dickinson, San Jose, CA, USA). The obtained raw data were further analyzed with software: Cylchred version 1.0.2 (University of Wales College of Medicine, Cardiff, UK) and Windows Multiple Document Interface version 2.8 (WinMDI 2.8) (http://facs.scripps.edu/software.html, accessed on 22 August 2021). All experiments were performed in duplicate and yielded similar results.

### 4.7. Analysis of Apoptosis by FACS Flow Cytometry

Determination of apoptosis was based on the detection of active caspase 3 in cancer cells via FACS flow cytometry, as previously described [156]. In brief, SK-N-AS and TE671 tumor cells were seeded on 6-well plates at a density of 1.0 × 10^6^ and 0.5 × 10^6^ cells per well, respectively, and left overnight to attach. Then, the culture medium was removed, and the cells were exposed to selected concentrations of ASBDs or DMSO. Camptothecin (20 µM) was used as a reference drug to induce apoptosis. After 24 h of treatment, the cells were harvested with accutase, centrifuged at 300× *g* for 5 min at +4 °C and washed twice with PBS. Then, a commercially available PE Active Caspase-3 Apoptosis Kit (BD Pharmingen™, BD Biosciences, San Diego, CA, USA) was used and the cells were incubated with phycoerythrin (PE)-conjugated anti-active caspase 3 antibody in accordance with the manufacturer’s protocols. Then, labeled tumor cells were analyzed using a flow cytometer FACSCalibur (Becton Dickinson, San Jose, CA, USA), operating with CellQuest software (Becton Dickinson, San Jose, CA, USA).

### 4.8. Protein Extraction and Western Blotting

Protein extraction and Western blotting were performed as previously described [157]. Briefly, whole cell protein lysates were prepared by scraping the cells in RIPA buffer (1% NP40, 0.5% sodium deoxycholate, 0.1% SDS, 1 mM EDTA, 1 mM EGTA, 1 mM Na_3_VO_4_, 20 mM NaF, 0.5 mM DTT, 1 mM PMSF and phosphatase and protease inhibitors cocktail) and centrifuged at 3000× *g* for 10 min at +4 °C. Total protein extracts were prepared in 3×Laemmli sample buffer (30% glycerol, 3% SDS, 0.19 M Tris-HCl, pH 6.8, 0.015% bromophenol blue and 3% *β*-mercaptoethanol), then boiled for 5 min at 100 °C. Equal amounts of protein extract were resolved via SDS-PAGE electrophoresis and electrotransfered (semi-dry system, Bio-Rad, Laboratories, Hercules, CA, USA) onto a polyvinylidene difluoride (PVDF) membrane. After blocking for 1 h at room temperature with 5% non-fat dry milk, membranes were probed overnight at +4 °C with following primary antibodies: anti-cleaved PARP1 (Asp214) rabbit pAb (#9541, Cell Signaling, Beverly, MA, USA); anti-cleaved caspase 3 (Asp175) rabbit pAb (#9661, Cell Signaling, Beverly, MA, USA); anti-phospho-Akt (D9E, Ser473) rabbit mAb (#4060, Cell Signaling, Beverly, MA, USA); anti-phospho-p44/42 MAPK (Erk1/2) (D13.14.4E, Thr202/Tyr204) rabbit mAb (#4370, Cell Signaling, Beverly, MA, USA); anti-phospho-p38 MAPK (3D7, Thr180/Tyr182) rabbit mAb (#9215, Cell Signaling, Beverly, MA, USA); anti-Akt1 (B-1) mouse mAb (sc-5298, Santa Cruz Biotechnology, Santa Cruz, CA, USA); anti-Erk2 (K-23) rabbit pAb (sc-153, Santa Cruz Biotechnology, Santa Cruz, CA, USA); anti-p38 *α*/*β* (H-147) rabbit pAb (sc-7149, Santa Cruz Biotechnology, Santa Cruz, CA, USA) diluted 1:1.000–1:2.000 in 5% BSA/TBS with 0.1% Tween-20. The next day, the membranes were incubated with relevant anti-rabbit or anti-mouse secondary antibody conjugated to horseradish peroxidase IgG-HRP (sc-2370 or sc-2371, respectively, Santa Cruz Biotechnology, Santa Cruz, CA, USA) diluted 1:2.000–1:5.000 in 1% BSA/TBS/0.1% Tween-20. Immunocomplexes were detected and visualized with enhanced chemiluminescence (Lumi-Light^PLUS^ Western Blotting Substrate, Roche Diagnostics GmbH, Mannheim, Germany) using the G:Box system and GeneTools analysis software (both from Syngene, Cambridge, UK). For stripping, membranes were incubated with stripping buffer (100 mM *β*-mercaptoethanol, 2% SDS, 62.5 mM Tris-HCl, pH 6.7) for 20 min at 50 °C, then washed, blocked, and re-probed with the relevant antibody, as described above. As a loading control for equal amounts of protein anti-*β*-actin (AC-15), mouse mAb (sc-69879, Santa Cruz Biotechnology, Santa Cruz, CA, USA) was used. Densitometric analysis was performed using ImageJ freeware software [158].

### 4.9. In Silico Analysis

The physicochemical properties of the analyzed ASBDs and Bet, including lipophilicity (n-octanol/water partition coefficient, LogP) and water solubility (LogS), were calculated in silico using ACD/ChemSketch Freeware software version 2021 1.0 (https://www.acdlabs.com/, accessed on 22 August 2021) and Swiss ADME tool (the SIB Swiss Institute of Bioinformatics, Molecular Modeling Group http://www.swissadme.ch/, accessed on 22 August 2021) [110]. The pharmacokinetic ADMET profile, including absorption, distribution, metabolism, excretion and toxicity, was predicted in silico using the admetSAR version 2.0 server (East China University of Science and Technology, School of Pharmacy, Shanghai Key Laboratory of New Drug Design, Laboratory of Molecular Modeling and Design http://lmmd.ecust.edu.cn/admetsar2, accessed on 22 August 2021) [76,77], whereas the druglikeness of ASBDs was evaluated using the Swiss ADME tool [110]. The prediction of potential target proteins and target pathways for Bet and ASBDs was computed with The Balanced Substructure–Drug–Target Network-Based Inference (bSDTNBI) method using NetInfer web server (http://lmmd.ecust.edu.cn/netinfer/, accessed on 22 August 2021). SDTNBI is the first network-based computational approach that can predict potential targets for new chemical agents on a large scale [79,80,81]. In all in silico studies, Bet was used as a reference compound. The chemical structures of Bet and both ASBDs were converted into canonical Simplified Molecular Input Line Entry Specification (SMILES) format using ACD/ChemSketch Freeware version 2021 1.0 software (Advanced Chemistry Development, Inc., Toronto, Ontario, Canada).

### 4.10. Statistical Analysis

All experiments were performed in at least 3 independent experiments (different cell passages) in duplicate or triplicate. The results are expressed as means ± standard error of the mean (SEM). *p* values were calculated using one-way or two-way analysis of variance (ANOVA), followed by Tukey’s or Dunnett’s or Sidak’s post hoc test for multiple comparisons. Statistical analyses were performed using GraphPad Prism version 6.0 (GraphPad Software, San Diego, CA, USA).

## 5. Conclusions

The biological findings from our in vitro study, including anti-survival, anti-proliferative and pro-apoptotic activities, along with ADMET profiling and filling the Lipinski druglikeness “rule-of-five”, all strongly suggest that the EB5 derivative may be considered as an interesting and promising anti-cancer compound. Although we are aware of several limitations of theoretical, in silico analyses, we believe that this kind of approach may be complementary to in vitro screening and might open new further research directions, and therefore will help in the development and optimization of Bet molecule-based drugs. Consequently, further studies are required to provide preclinical and clinical validations of ASBDs.

## Figures and Tables

**Table 1:** Cytotoxicity of CDDP, TMZ and ASBDs by means of IC_50_ values.

Cell Line	IC_50_				
	CDDP	TMZ	EB5	EB25/1	Bet
SK-N-AS	3.22 µM	486.59 µM	0.62 µM	18.77 µM	2.5 µM [18]
TE671	2.48 µM	714.48 µM	1.34 µM	17.35 µM	10.3 µM [18]
HSF	– ^1^	–	17.15 µM	N/A ^2^	–

^1^—not tested; ^2^ N/A—not applicable in tested concentrations.

**Table 2:** Physicochemical parameters of Bet and ASBDs—comparison of values obtained both experimentally and in silico.

	Compound	Bet	EB5	EB25/1
Parameter				
Formula		C_30_H_50_O_2_	C_33_H_50_O_3_	C_34_H_52_O_4_
Molecular Weight [g/mol]		442.72	494.75	524.77
Lipophilicity				
LogP_TLC_		5.41 [61]	7.08 [61]	7.76 [61]
LogP (iLOGP ^1^)		4.47	5.11	5.44
LogP (XLOGP3 ^2^)		8.28	9.34	9.38
LogP (WLOGP ^3^)		7.00	7.26	7.87
LogP (MLOGP ^4^)		6.00	6.29	6.03
LogP (SILICOS-IT ^5^)		6.21	6.96	6.92
Consensus LogP ^6^		6.39	6.99	7.13
Water Solubility				
LogS (ESOL ^7^)		−7.67	−8.53	−8.61
Solubility		9.48 × 10^−6^ mg/mL; 2.14 × 10^−8^ mol/l	1.47 × 10^−6^ mg/mL; 2.97 × 10^−9^ mol/l	1.30 × 10^−6^ mg/mL; 2.47 × 10^−9^ mol/l
Class ^8^		Poorly soluble	Poorly soluble	Poorly soluble
LogS (Ali ^9^)		−8.99	−10.22	−10.46
Solubility		4.50 × 10^−7^ mg/mL; 1.02 × 10^−9^ mol/l	2.98 × 10^−8^ mg/mL; 6.02 × 10^−11^ mol/l	1.84 × 10^−8^ mg/mL; 3.50 × 10^−11^ mol/l
Class		Poorly soluble	Insoluble	Insoluble
LogS (SILICOS-IT ^10^)		−6.17	−6.46	−6.57
Solubility		2.99 × 10^−4^ mg/mL; 6.75 × 10^−7^ mol/l	1.70 × 10^−4^ mg/mL; 3.44 × 10^−7^ mol/l	1.40 × 10^−4^ mg/mL; 2.67 × 10^−7^ mol/l
Class		Poorly soluble	Poorly soluble	Poorly soluble

^1^ iLOGP—in house physics-based method implemented from [62]. ^2^ XLOGP3—atomistic and knowledge-based method calculated by XLOG software, version 3.2.2 [63]. ^3^ WLOGP—atomistic method implemented from [64]. ^4^ MLOGP—topological method implemented from [65,66,67].^5^ SILICOS-IT—hybrid fragmental/topological method calculated by FILTER-IT program, version 1.0.2 from [68]. ^6^ Consensus LogP—the average of all 5 computational predictions. ^7^ LogS ESOL—estimated SOLubility, topological method implemented from [69]. ^8^ Class—LogS scale: insoluble < −10<poorly < −6<moderately < −4 < soluble < −2 < very < 0 < highly. ^9^ LogS Ali—topological method implemented from [70]. ^10^ LogS SILICOS-IT—fragmental method calculated by FILTER-IT program, version 1.0.2 from [68].

**Table 3:** Druglikeness of Bet and ASBDs—in silico analysis.

	Compound	Bet	EB5	EB25/1
Violation				
Lipinski ^1^		Yes; 1 violation: MLOGP > 4.15	Yes; 1 violation: MLOGP > 4.15	No; 2 violations: MW > 500, MLOGP > 4.15
Ghose ^2^		No; 3 violations: WLOGP > 5.6, MR > 130, #atoms > 70	No; 4 violations: MW > 480, WLOGP > 5.6, MR > 130, #atoms > 70	No; 4 violations: MW > 480, WLOGP > 5.6, MR > 130, #atoms > 70
Veber ^3^		Yes	Yes	Yes
Egan ^4^		No; 1 violation: WLOGP > 5.88	No; 1 violation: WLOGP > 5.88	No; 1 violation: WLOGP > 5.88
Muegge ^5^		No; 1 violation: XLOGP3 > 5	No; 1 violation: XLOGP3 > 5	No; 1 violation: XLOGP3 > 5
Bioavailability Score ^6^		0.55	0.55	0.17

^1^ Lipinski guidelines—implemented from [67] MW ≤ 500, MLOGP ≤ 4.15, N or O ≤ 10, NH or OH ≤ 5. ^2^ Ghose guidelines—implemented from [71] 160 ≤ MW ≤ 480, −04. ≤ WLOGP ≤ 5.6, 40 ≤ MR ≤ 130, 20 ≤ atoms ≤ 70. ^3^ Veber guidelines—implemented from [72] rotatable bond ≤ 10, TPSA ≤ 140. ^4^ Egan guidelines—implemented from [73] WLOG ≤ 5.88, TPSA ≤ 131.6. ^5^ Muegge guidelines—implemented from [74] 200 ≤ MW ≤ 600, −2 ≤ XLOGP ≤ 5, TPSA ≤1 50, num. rings ≤ 7, num. carbon > 4, num. heteroatoms > 1, num. of rotatable bonds ≤ 15, H-BA ≤ 10, H-BD ≤ 5. ^6^ Bioavailability—implemented from [75] probability of drug oral bioavailability (F) > 10% in rat.

**Table 4:** Pharmacokinetic parameters of Bet and ASBDs—computational predictions of ADMET profile.

	Compound	Bet		EB5		EB25/1	
Parameter							
ADMET Profile Classifications		Value	Probability	Value	Probability	Value	Probability
Absorption							
HIA ^1^		+	0.9884	+	0.9892	+	0.9818
Caco-2 permeability		−	0.5542	−	0.6911	−	0.7424
Human oral bioavailability		−	0.5857	−	0.6571	−	0.6714
Distribution							
Subcellular localization		Lys ^2^	0.4831	Mito ^3^	0.8480	Mito	0.8300
BBB ^4^ permeant		−	0.4533	+	0.8120	+	0.9081
P-glycoprotein inhibitor		−	0.8836	−	0.7952	−	0.4746
P-glycoprotein substrate		−	0.7347	−	0.8347	−	0.6175
BSEP ^5^ inhibitor		+	0.6370	+	0.8859	+	0.7854
OATP ^6^ 1B1 inhibitor		+	0.9413	+	0.9013	+	0.9004
OATP 1B3 inhibitor		+	0.9480	+	0.8936	+	0.8682
OATP 2B1 inhibitor		−	0.7184	−	0.7112	−	0.5653
OCT2 ^7^ inhibitor		−	0.6385	−	0.6000	−	0.6526
MATE1 ^8^ inhibitor		−	1.0000	−	0.8200	−	0.9600
Metabolism							
CYP450 ^9^ 3A4 substrate		+	0.6751	−	0.6453	+	0.7183
CYP450 2C9 substrate		−	0.6284	−	0.5974	+	0.5886
CYP450 2D6 substrate		−	0.7222	−	0.5760	−	0.7448
CYP450 1A2 inhibition		−	0.9045	−	0.9286	−	0.8561
CYP450 2C9 inhibition		−	0.9071	−	0.8779	−	0.7006
CYP450 2C19 inhibition		−	0.9026	−	0.6899	−	0.6710
CYP450 2D6 inhibition		−	0.9297	−	0.6000	−	0.9188
CYP450 3A4 inhibition		−	0.8309	−	0.9281	−	0.6587
CYP inhibitory promiscuity		−	0.6441	−	0.6416	−	0.7258
Toxicity							
Carcinogenicity		−	0.9857	−	0.8347	−	0.9073
Ames mutagenesis		−	0.7500	−	0.7000	−	0.6250
Eye corrosion		−	0.9892	−	0.9923	+	0.7267
Eye irritation		−	0.9008	−	0.9001	−	0.7900
Hepatotoxicity		−	0.6250	−	0.5500	+	0.7678
Acute Oral Toxicity		III ^10^	0.7441	III	0.6655	−	0.5236

Machine learning methods, including support vector machine (SVM), random forest (RF), k-nearest neighbors (k-NN) and deep learning methods, such as convolutional neural network (CNN) implemented for building ADMET qualitative classifications models described in [76,77]. ^1^ HIA—human intestinal absorption; ^2^ Lys—lysosome; ^3^ Mito—mitochondria; ^4^ BBB—blood–brain barrier; ^5^ BSEP—bile salt export pump; ^6^ OATP—organic anion-transporting polypeptide; ^7^ OCT2—organic cation transport protein 2; ^8^ MATE1—multidrug and toxin extrusion transporter 1; ^9^ CYP450—cytochrome P450; ^10^ III—category of toxicity according to the U.S. Environmental Protection Agency (EPA) [78].

**Table 5:** Prediction of target proteins for ASBDs—computational approach.

Compound	Target ID ^1^	Protein Name ^2^	Protein Family	Gene Symbol ^3^	Gene ID ^4^	Organism	Score
Bet	P04278	Sex hormone-binding globulin	-	SHBG	6462	*Homo sapiens* (Human)	0.000334142
	P34972	Cannabinoid receptor 2 (CB-2)	G protein coupled receptor 1 family	CNR2	1269		0.000277126
	Q8TDU6	G protein coupled bile acid receptor 1		GPBAR1	151306		0.000266207
	P04150	Glucocorticoid receptor (GR)	Nuclear hormone receptor family	NR3C1	2908		0.000243245
	P41145	Kappa-type opioid receptor (K-OR-1)	G protein coupled receptor 1 family	OPRK1	4986		0.000236339
	P41143	Delta-type opioid receptor (D-OR-1)		OPRD1	4985		0.000213809
	P35372	Mu-type opioid receptor (M-OR-1)		OPRM1	4988		0.000201019
	P32249	Epstein–Barr virus-induced molecule 2 (G protein coupled receptor 183)		GPR183	1880		0.000196558
	Q9NUW8	Tyrosyl-DNA phosphodiesterase 1	Tyrosyl-DNA phosphodiesterase family	TDP1	55775		0.000188861
	P03372	Estrogen receptor (ER-*α*)	Nuclear hormone receptor family	ESR1	2099		0.000188836
EB5	P04278	Sex hormone-binding globulin	-	SHBG	6462	*Homo sapiens* (Human)	0.000271256
	P41594	Metabotropic glutamate receptor 5 (mGluR5)	G protein coupled receptor 3 family	GRM5	2915		0.000224581
	P04150	Glucocorticoid receptor (GR)	Nuclear hormone receptor family	NR3C1	2908		0.000220946
	P04035	3-hydroxy-3-methylglutaryl-coenzyme A reductase (HMG-CoA reductase)	HMG-CoA reductase family	HMGCR	3156		0.000208677
	P41145	Kappa-type opioid receptor (K-OR-1)	G protein coupled receptor 1 family	OPRK1	4986		0.00020737
	P34972	Cannabinoid receptor 2 (CB-2)		CNR2	1269		0.000204833
	Q9NUW8	Tyrosyl-DNA phosphodiesterase 1	Tyrosyl-DNA phosphodiesterase family	TDP1	55775		0.00019616
	Q8TDU6	G protein coupled bile acid receptor 1	G protein coupled receptor 1 family	GPBAR1	151306		0.00018902
	P41143	Delta-type opioid receptor (D-OR-1)		OPRD1	4985		0.000166818
	Q16236	Nuclear factor erythroid 2-related factor 2 (NRF2)	BZIP family	NFE2L2	4780		0.000160777
EB25/1	P04278	Sex hormone-binding globulin	-	SHBG	6462	*Homo sapiens* (Human)	0.000268805
	P04150	Glucocorticoid receptor (GR)	Nuclear hormone receptor family	NR3C1	2908		0.000226021
	P41594	Metabotropic glutamate receptor 5 (mGluR5)	G protein coupled receptor 3 family	GRM5	2915		0.000225983
	Q9NUW8	Tyrosyl-DNA phosphodiesterase 1	Tyrosyl-DNA phosphodiesterase family	TDP1	55775		0.000223637
	P34972	Cannabinoid receptor 2 (CB-2)	G protein coupled receptor 1 family	CNR2	1269		0.000205072
	P41145	Kappa-type opioid receptor (K-OR-1)		OPRK1	4986		0.000200803
	P04035	3-hydroxy-3-methylglutaryl-coenzyme A reductase (HMG-CoA reductase)	HMG-CoA reductase family	HMGCR	3156		0.000190366
	Q8TDU6	G protein coupled bile acid receptor 1	G protein coupled receptor 1 family	GPBAR1	151306		0.000186738
	P20309	Muscarinic acetylcholine receptor M3 (mAChR M3)		CHRM3	1131		0.000169216
	P41143	Delta-type opioid receptor (D-OR-1)		OPRD1	4985		0.000162948

^1^ Target ID and ^2^ Protein name—according to the UniProtKB/Swiss-Prot database. ^3^ Gene symbol and ^4^ Gene ID—according to the NCBI Entrez Gene database. Method: Balanced Substructure–Drug–Target Network-Based Inference (bSDTNBI); implemented from [79,80,81]. Network: global drug–target interaction (DTI) network (version 2020). Molecular fingerprint: implemented from Klekota-Roth (KR) [59]. Other parameters: *α* = 0.1, *β* = 0.1, *γ* = −0.5, k = 2, top 10; default settings implemented from [82].

**Table 6:** Prediction of target pathways for ASBDs—in silico analysis.

Compound	Pathway ID ^1^	Description	Score
Bet	hsa04610	Complement and coagulation cascades	0.000706300
	hsa04510	Focal adhesion	0.000645716
	hsa04151	PI3K-Akt signaling pathway	0.000573224
	hsa04915	Estrogen signaling pathway	0.000562535
	hsa03320	PPAR signaling pathway	0.000518514
	hsa04974	Protein digestion and absorption	0.000484971
	hsa04978	Mineral absorption	0.000438870
	hsa04216	Ferroptosis	0.000430336
	hsa04640	Hematopoietic cell lineage	0.000409757
	hsa04115	p53 signaling pathway	0.000404527
EB5	hsa04610	Complement and coagulation cascades	0.000683879
	hsa04510	Focal adhesion	0.000611875
	hsa04151	PI3K-Akt signaling pathway	0.000543412
	hsa04915	Estrogen signaling pathway	0.000542487
	hsa03320	PPAR signaling pathway	0.000497120
	hsa04974	Protein digestion and absorption	0.000470290
	hsa04216	Ferroptosis	0.000432794
	hsa04978	Mineral absorption	0.000420103
	hsa04640	Hematopoietic cell lineage	0.000407986
	hsa04115	p53 signaling pathway	0.000386938
EB25/1	hsa04610	Complement and coagulation cascades	0.000659049
	hsa04510	Focal adhesion	0.000598057
	hsa04915	Estrogen signaling pathway	0.000535527
	hsa04151	PI3K-Akt signaling pathway	0.000531424
	hsa03320	PPAR signaling pathway	0.000482445
	hsa04974	Protein digestion and absorption	0.000455536
	hsa04216	Ferroptosis	0.000423241
	hsa04978	Mineral absorption	0.000419440
	hsa04640	Hematopoietic cell lineage	0.000393377
	hsa04115	p53 signaling pathway	0.000373228

^1^ Pathway ID—according to the KEGG PATHWAYS database [83,84]. Method: Balanced Substructure–Drug–Target Network-Based Inference (bSDTNBI) implemented from [79,80,81]. Network: drug-pathway association network for pan-cancer (version 2020). Molecular fingerprint: implemented from Klekota-Roth (KR) [59]. Other parameters: *α* = 0.1, *β* = 0.1, *γ* = -0.5, k = 2, top 10; default settings implemented from [82].

## Supplementary Materials

The following are available online at https://www.mdpi.com/article/10.3390/ijms222212299/s1. Figure S1: ^1^H-NMR spectrum for EB5 (600 MHz, CDCl_3_), Figure S2: ^13^C-NMR spectrum for EB5 (150 MHz, CDCl_3_), Figure S3: ^1^H-NMR spectrum for EB25/1 (600 MHz, CDCl_3_), Figure S4: ^13^C-NMR spectrum for EB25/1 (150 MHz, CDCl_3_).
