# Supplementary material for: Acetylenic Synthetic Betulin Derivatives Inhibit Akt and Erk Kinases Activity, Trigger Apoptosis and Suppress Proliferation of Neuroblastoma and Rhabdomyosarcoma Cell Lines
Source: Int J Mol Sci. 2021 Nov 14;22(22):12299. doi: 10.3390/ijms222212299 (PMC8624615; doi:10.3390/ijms222212299)
Supplement: Supplementary file 1 [file ijms-22-12299-s001.zip › ijms-1452123-supplementary.pdf]

Supplementary Materials

Acetylenic Synthetic Betulin Derivatives Inhibit Akt and Erk Kinases Activity, Trigger Apoptosis and Suppress Proliferation of Neuroblastoma and Rhabdomyosarcoma Cell Lines

Sylwia K. Król [1],* Ewa Bębenek [2], Magdalena Dmoszyńska-Graniczka [1], Adrianna Sławińska-Brych [3], Stanisław Boryczka [2] and Andrzej Stepulak [1]

[1] Department of Biochemistry and Molecular Biology, Faculty of Medicine, Medical University of Lublin, Chodźki 1, 20-093 Lublin, Poland; magdalena.dmoszynska-graniczka@umlub.pl (M.D.-G.); andrzej.stepulak@umlub.pl (A.S.)
[2] Department of Organic Chemistry, Faculty of Pharmaceutical Sciences in Sosnowiec, Medical University of Silesia, Jagiellońska 4, 41-200, Sosnowiec, Poland; ebebenek@sum.edu.pl (E.B.); boryczka@sum.edu.pl (S.B.)
[3] Department of Cell Biology, Faculty of Biology and Biotechnology, Institute of Biological Sciences, Maria Curie-Sklodowska University, Akademicka 19, 20-033 Lublin, Poland; adrianna.slawinska-brych@poczta.umcs.lublin.pl
* Correspondence: sylwia_krol15@wp.pl

LIST OF CONTENTS

Figure S1. ^{1}H-NMR spectrum for EB5 (600 MHz, $CDCl_3$)

Figure S2. ^{13}C-NMR spectrum for EB5 (150 MHz, $CDCl_3$)

Figure S3. ^{1}H-NMR spectrum for EB25/1 (600 MHz, $CDCl_3$)

Figure S4. ^{13}C-NMR spectrum for EB25/1 (150 MHz, $CDCl_3$)

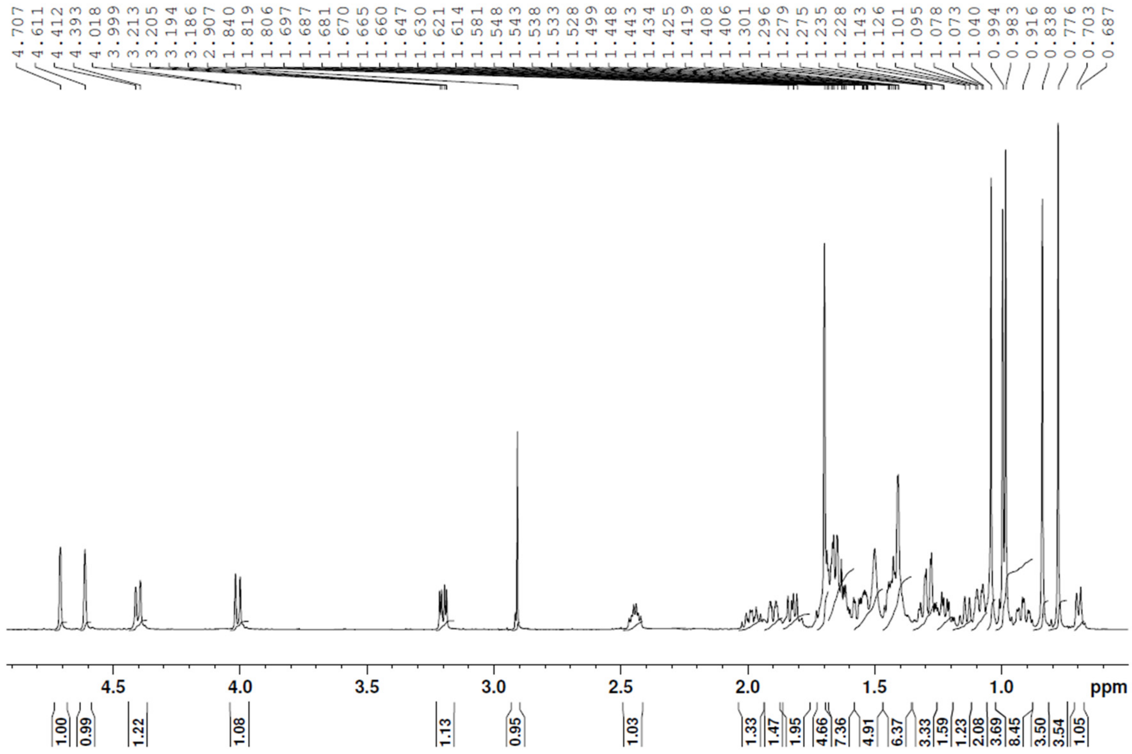

Figure S1. ^{1}H-NMR spectrum for EB5 (600 MHz, $CDCl_3$)

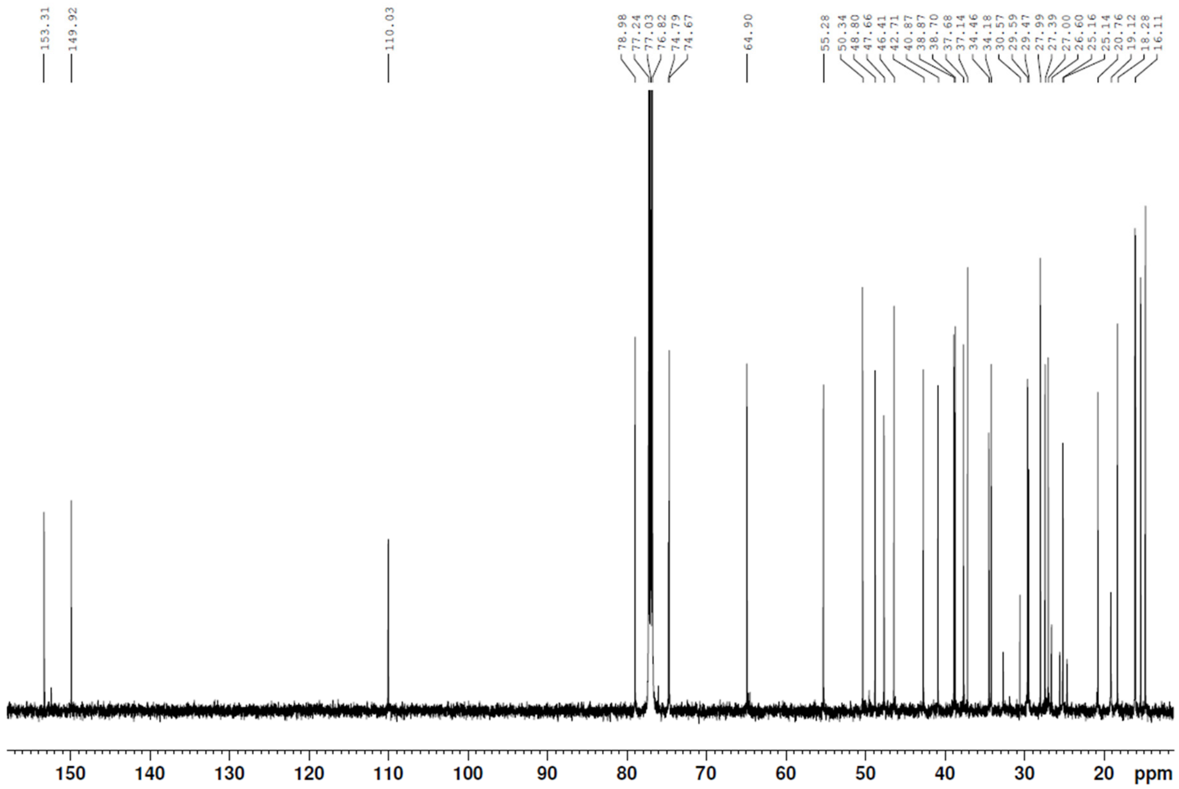

FigureS2. ^{13}C-NMR spectrum for EB5 (150 MHz, $CDCl_3$)

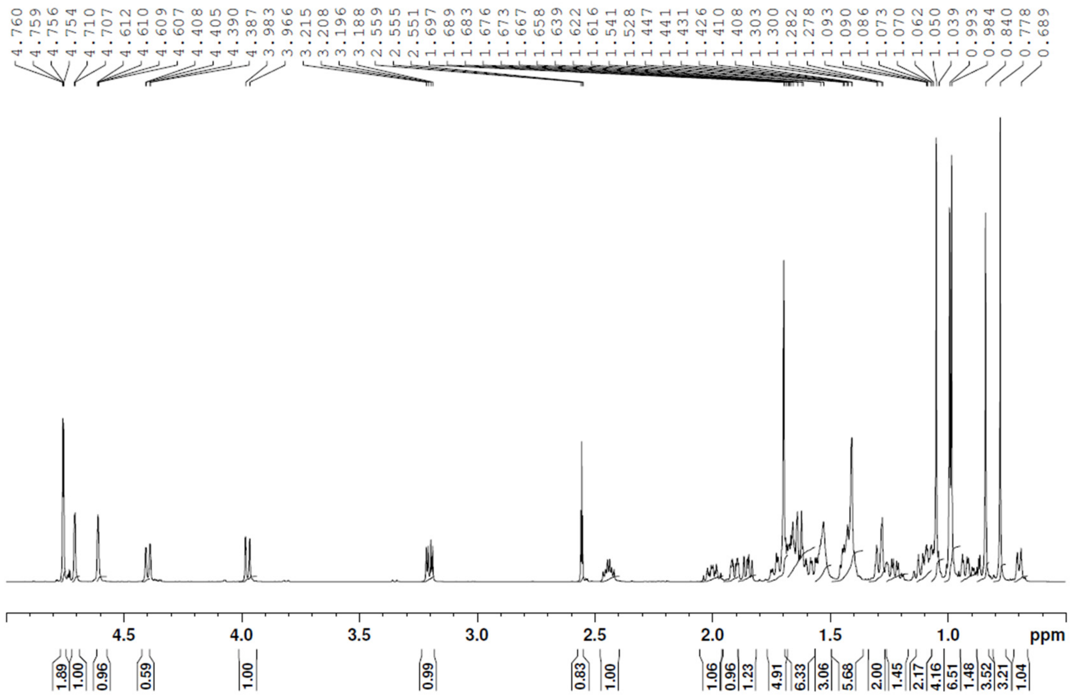

Figure S3. ^{1}H-NMR spectrum for EB25/1 (600 MHz, $CDCl_3$)

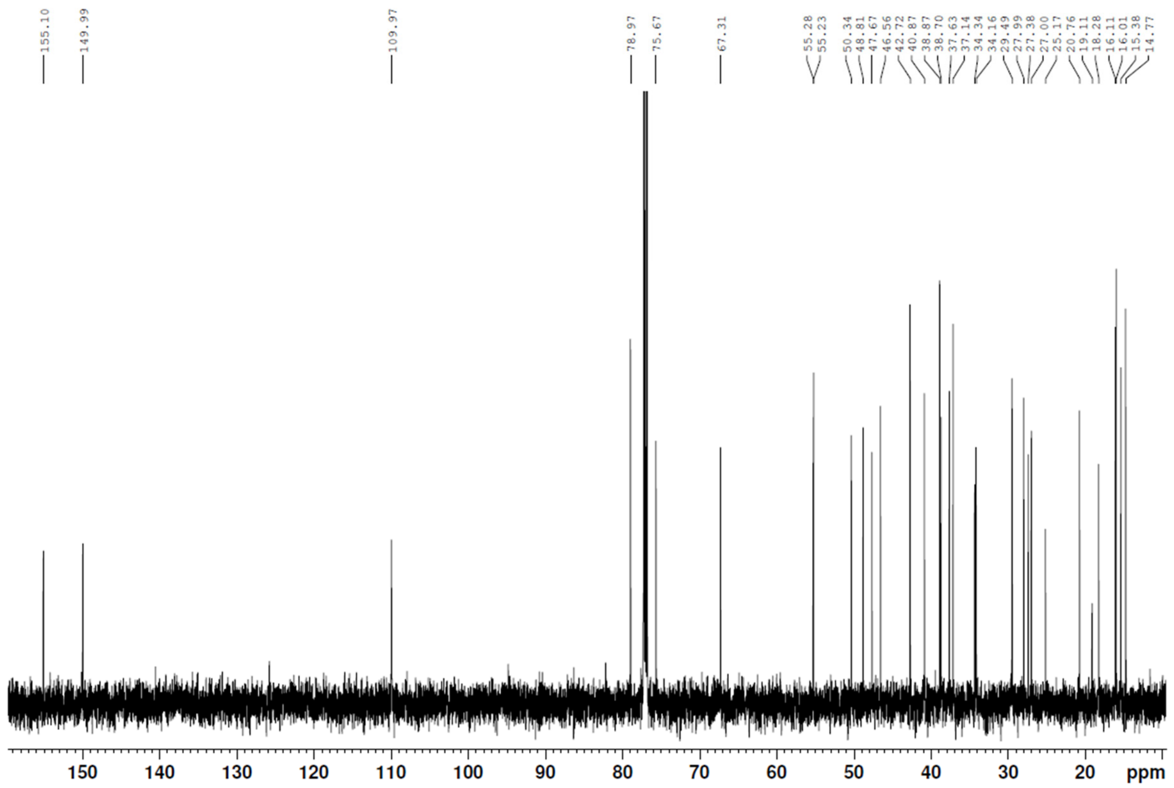

Figure S4. ^{13}C-NMR spectrum for EB25/1 (150 MHz, $CDCl_3$)
